# P-1145. Resistance to TMP-SMX in Stenotrophomonas maltophilia: Evidence from Guatemala

**DOI:** 10.1093/ofid/ofaf695.1339

**Published:** 2026-01-11

**Authors:** Kenia Algaba, Diego Erdmenger, Sara Estrada, Luis Rodriguez

**Affiliations:** Hospital General San Juan de Dios, Guatemala, El Progreso, Guatemala; Hospital General San Juan de Dios, Guatemala, El Progreso, Guatemala; Hospital General San Juan de Dios, Guatemala, El Progreso, Guatemala; Hospital General San Juan de Dios, Guatemala, El Progreso, Guatemala

## Abstract

**Background:**

*Stenotrophomonas maltophilia* (SM) is an emerging opportunistic pathogen of increasing clinical relevance due to its intrinsic multidrug resistance and the associated risk of therapeutic failure. Infections caused by SM are frequently observed in intensive care unit (ICU) populations.

Its global prevalence has been rising, with reported rates of 32% in Europe and 27.7% in the Americas. Resistance to trimethoprim-sulfamethoxazole (TMP-SMX), the first-line therapy for SM, has been reported in up to 13% of isolates in the Americas. However, data on the prevalence and resistance patterns of SM in hospitalized patients in Guatemala remain unavailable.

**Methods:**

An ecological analysis of all *Stenotrophomonas maltophilia* isolates collected between 2022 and 2024 from a Guatemalan national hospital was conducted. The analysis included the site of sample isolation and the hospital unit from which each isolate originated. Resistance to trimethoprim-sulfamethoxazole (TMP-SMX) was determined according to Clinical and Laboratory Standards Institute criteria (≥4/76 mg/L).

**Results:**

A total of 349 *Stenotrophomonas maltophilia* isolates were identified during the study period. An increase in SM isolates was observed in 2023 and 2024 compared to 2022 (84 in 2022, 153 in 2023, and 112 in 2024). The majority of isolates were recovered from endotracheal aspirates (63.32%), followed by blood cultures (10.02%). Nearly half of all isolates originated from patients in the intensive care unit (ICU) (n = 170; 48.71%), followed by the Emergency Department (n = 90; 28.36%).

Overall, resistance to trimethoprim-sulfamethoxazole (TMP-SMX) was identified in 65 isolates (18.84%). A higher rate of TMP-SMX resistance was observed in isolates from endotracheal aspirates (19.9%) compared to blood cultures (5.71%) and urine cultures (16.66%). There was no statistically significant difference in resistance rates based on the hospital unit of origin, with ICU samples showing 22.35% resistance, Emergency Department samples 15.15%, and inpatient ward samples 15%.

**Conclusion:**

*Stenotrophomonas maltophilia* shows increasing prevalence and notable resistance to TMP-SMX, particularly in ICU respiratory samples, underscoring the need for targeted surveillance and stewardship in high-risk hospital settings.

**Disclosures:**

All Authors: No reported disclosures

